# Identification of Potential Biomarkers and Spectral Fingerprinting for Detection of Foodborne Pathogens in Raw Chicken Meat Matrix Using GCMS and FTIR

**DOI:** 10.3390/foods13213416

**Published:** 2024-10-26

**Authors:** Gayathri Muthusamy, Subburamu Karthikeyan, Veeranan Arun Giridhari, Ahmad R. Alhimaidi, Dananjeyan Balachandar, Aiman A. Ammari, Vaikuntavasan Paranidharan, Thirunavukkarasu Maruthamuthu

**Affiliations:** 1 Department of Agricultural Microbiology, Tamil Nadu Agricultural University, Coimbatore 641003, India; gayathri23997@gmail.com (G.M.); dbalu@tnau.ac.in (D.B.); 2 Centre for Post Harvest Technology, Agricultural Engineering College and Research Institute, Tamil Nadu Agricultural University, Coimbatore 641003, India; vag@tnau.ac.in; 3 Department of Zoology, College of Science, King Saud University, Riyadh 11451, Saudi Arabia; 4 Department of Plant Pathology, Tamil Nadu Agricultural University, Coimbatore 641003, India; agriparani@yahoo.com; 5 Department of Agronomy, Tamil Nadu Agricultural University, Coimbatore 641003, India; thirunavukkarasu.m@tnau.ac.in

**Keywords:** foodborne pathogens, meat metabolites, spectral fingerprinting, biomarker, meat

## Abstract

Foodborne illnesses pose a serious threat to public health, with increasing global incidence rates driven by factors such as rising meat consumption. Rapid detection of foodborne pathogens in meat is critical for preventing outbreaks. This study investigates the potential of gas chromatography-mass spectrometry (GC-MS) and Fourier-transform infrared spectroscopy (FTIR) for identifying biomarkers and spectral fingerprints indicative of foodborne pathogens in raw chicken meat. Raw broiler chicken meat samples were surface-sterilized and inoculated with foodborne pathogens. The samples were challenge inoculated with the specific pathogen and the physical quality parameters like pH, color, texture, drip loss, and water activity were assessed. GC-MS analysis identified 113 metabolites, including potential biomarkers like ureidopropionic acid, 5-sulfosalicylic acid, 11,14-eicosadienoic acid, methyl ester for *E*. *coli* O157:H7; 11-bromoundecanoic acid, neocurdione, glafenin, eicosanoic acid for *Salmonella*; azepan-1-yl-acetic acid, methyl ester, tramadol, cytarabine, dipipanone for *Staphylococcus* and cyclopentaneundecanoic acid, phosphonofluoridic acid, î-n-formyl-l-lysine for *Pseudomonas*. Pathway analysis revealed the involvement of fatty acid metabolism and amino acid degradation pathways. FTIR spectral data showed significant variances between control and spiked samples, particularly in the fatty acid spectral region. The identified metabolites and spectral patterns could serve as biomarkers for developing rapid pathogen detection methods, contributing to enhanced food safety protocols.

## 1. Introduction

Foodborne illness continues to pose a significant global health burden. According to the World Health Organization (WHO), nearly one in ten people fall ill each year due to foodborne pathogens, with 33 million healthy life years lost. The global incidence of foodborne illnesses stands at 600 million cases annually, resulting in 420,000 deaths. Particularly vulnerable are children under the age of five, who account for 30% of all foodborne disease-related deaths. These alarming statistics underscore the urgent need for improved food safety measures worldwide.

High-profile outbreaks, such as the *Salmonella* outbreaks linked to Italian-style meats (CDC, 2022) and ground beef, have attracted media attention and raised public awareness of the risks associated with consuming contaminated food. Notable foodborne pathogens like *Salmonella*, *Escherichia coli*, and *Listeria* have been at the forefront of these outbreaks. In response to increasing concerns for public health, food safety has become a global priority, with a heightened focus on the prevention of pathogen-related contamination.

Meat, as a major source of protein in diets globally, is a central focus in discussions of food safety. The Organization for Economic Co-operation and Development (OECD) and the Food and Agriculture Organization of the United Nations (FAO) have reported that meat consumption has doubled over the past five decades, with a projected 14% increase by 2030 due to population growth. Poultry is expected to contribute to 41% of total global meat protein, followed by pig meat (34%), beef (20%), and sheep meat (5%). This projected increase in meat consumption heightens the need for rapid, accurate detection of foodborne pathogens to prevent outbreaks and ensure public health.

Conventional culture-based detection methods of foodborne pathogens, though considered the gold standard, are often time-consuming, leading to delays that compromise food safety. Culture-based detection is a progressive approach to cultural enrichment that includes strain typing, confirmation, and selective and differential plating. Enrichment restores damaged cells and raises the target pathogen concentration in food samples, and selective enrichment increases the concentration of a certain pathogen in food samples by using particular media. The environment, temperature, incubation period, and different nutrients in the media all affect the culture’s state [1]. Conventional methods are time-consuming, less sensitive, and labor-intensive. Recent advances in analytical techniques, such as GCMS and FTIR, offer powerful tools for identifying biomarkers indicative of foodborne pathogens in complex matrices like meat. These methods allow for more rapid and precise detection, fostering greater consumer confidence in the safety of food products [2,3,4,5].

GCMS, in particular, has shown immense potential in detecting foodborne pathogens by identifying specific metabolites or biomarkers produced by pathogens during meat contamination. For example, one study demonstrated that GCMS could detect Salmonella and *E*. *coli* at concentrations as low as 7 CFU/25 g of a food sample [6]. Furthermore, researchers have used GCMS to identify metabolites and volatile organic compounds (VOCs) produced by *Salmonella typhimurium*, *Campylobacter jejuni*, and *Staphylococcus aureus* in different types of raw meat, including beef, pork, and chicken [7]. Another study employed solid-phase microextraction (SPME) probes to detect untargeted microbial metabolites on meat matrices [8]. Microorganisms have been identified and categorized according to their unique metabolic properties using (FT-IR) spectroscopy in conjunction with multivariate statistical methods [9]. In a study conducted by Xu et al. [10], the use of reflectance FTIR microscopic imaging for rapid, non-invasive detection and classification of *Bacillus subtilis* and *Escherichia coli* dried on stainless steel and aluminum substrates was explored. This research outcome highlights FTIR’s potential for characterizing bacterial spectroscopic fingerprints in food processing environments. Each bacterial cell produces a unique absorption spectrum through FTIR, representing its distinct fingerprint signature and reflecting its specific biomolecular composition [11]. The FTIR technique is well-suited for routine quality control or industrial applications, offering a high level of confidence in its results [12].

The emerging field of meat metabolomics offers a valuable approach in understanding the nutritional quality, safety, and authenticity of meat products. Through the analysis of small molecules present in meat, metabolomics provides insight into the role of metabolites in meat quality and safety. In addition to GC-MS and FTIR, several advanced techniques have emerged for detecting foodborne pathogens, including biosensors, Raman spectroscopy, and CRISPR-based methods. While GC-MS and FTIR are powerful tools that provide detailed metabolite profiling and molecular fingerprinting, newer technologies such as electrochemical biosensors with nanomaterials offer rapid diagnostics. These biosensors, which utilize quantum dots, nanoparticles, and carbon nanomaterials, enhance sensitivity and speed [13]. A fiber-optic portable Raman probe, combined with machine learning, allows real-time detection of pathogen-specific volatile organic compounds (VOCs) with high accuracy, even at significant dilutions [14]. Additionally, the CRISPR/Cas12a-mediated CSDHCR nucleic acid assay provides ultrasensitive, rapid, and visual detection of pathogens, with a detection limit of 4.54 fM. The CRISPR-based assay allows for quicker, colorimetric detection with excellent specificity, making it a promising alternative for practical food safety applications [15].

The current study focuses on the identification of biological markers (metabolites) using GCMS and spectral fingerprinting of raw meat matrixes to detect foodborne pathogens. Unlike CRISPR, which is limited to genetic detection, and biosensors, which often need pathogen-specific markers, FTIR and GC-MS can be applied to various ranges of compound identification, providing a more universal detection method. Also, GC-MS excels at detecting even trace amounts of metabolites produced by pathogens, while FTIR is sensitive to chemical bond changes induced by foodborne pathogens. While highly specific, biosensors and CRISPR may not detect metabolic byproducts, limiting their utility in cases where metabolic shifts indicate the presence of foodborne pathogens. The high-throughput capabilities of metabolomics platforms enhance the scalability and efficiency of food safety protocols, addressing the challenges posed by modern food production systems.

## 2. Materials and Methods

### 2.1. Raw Meat Sample

Raw broiler chicken meat sample was collected (two biological replications were maintained) in a sterile bag from a local butcher shop (11.001987° latitude and 76.948374° longitude) at Coimbatore, Tamil Nadu, India, transported to the laboratory in an ice box at 4 °C, and analyzed immediately.

#### 2.1.1. Inoculum Preparation

The reference strains used in the study included *Escherichia coli* O157:H7 STEC strain (O157-TNAU), *Staphylococcus aureus* (ATCC 25923), *Pseudomonas aeruginosa* PAO1 (cultures obtained from TNAU repository), and *Salmonella typhimurium* (a clinical isolate obtained from SRM University). These bacterial cultures were streaked onto sterile nutrient agar plates and grown for 24 ± 2 h at 37 ± 2 °C. The purity of the reference strains was confirmed by performing PCR assay with species-specific primers of the individual pathogen (Supplementary Table S3). After this, the plates were stored at 4 °C. During the experiment, a single colony from the pure cultures of each bacterial inoculum was inoculated to sterile LB broth and incubated for 24 ± 2 h under shaking at 37 ± 2 °C. Following this, the bacterial concentration was optimized to 6 log CFU/mL by reading the optical density (OD) at 600 nm.

#### 2.1.2. Spiking on Meat Matrix

Raw broiler chicken meat was spiked with respective microbial cultures following the method of Cevallos-Cevallos et al. [6]. Samples of two biological replicates of approximately 500 g each were obtained, surface sterilized individually with 70% ethyl alcohol, and allowed to air-dry under aseptic conditions. These two biological replicates were made into three technical replicates. All the above-mentioned bacterial cultures, approximately at 6 log CFU/mL were individually inoculated to the surface sterilized meat sample (150 g each) contained in a sterile glass beaker. The bacterial inoculum was spiked to the meat samples using a sterile pipette, followed by gentle shaking. Aliquots were taken from different areas to verify uniform distribution before further analysis. An uninoculated raw meat matrix was maintained as a control. All were then incubated at 37 ± 2 °C for 2 h. The microbial load of the spiked pathogens was counted individually after incubation time.

### 2.2. Meat Quality Parameters

The quality parameters of raw chicken meat such as pH, color, texture, drip loss, and water activity were observed as follows.

#### 2.2.1. Determination of pH

The pH of the collected raw meat sample was recorded as determined by Lee et al. [16]. In total, 5 g of the raw meat sample was blended with 45 mL of distilled water and after 5 min the pH (pH meter, Ohaus ST-3100F, Parispanny, NJ, USA) was observed using a glass electrode.

#### 2.2.2. Color Profiling of Raw Chicken Meat

The color of the raw chicken meat was observed by a tintometer (Lovibond LC 100, India) instrument expressed as lightness (L*), redness (a*), and yellowness (b*) [17]. Prior to observation, the instrument was calibrated using a white plate. An area of 50 mm, illuminant D65, and 2° standard observer were used.

#### 2.2.3. Texture Profiling of Raw Chicken Meat

Raw chicken meat texture profiling was carried out using a texture analyzer (TA-HD Plus Texture Analyzer, New Castle, DE, USA). Using a cylindrical probe of P36/R, meat samples measuring 1.0 × 1.0 × 1.0 cm were positioned parallel on a flat metal plate, with the muscle fiber axis perpendicular to the probe’s movement [18]. Primary factors such as cohesion, adhesiveness, springiness, hardness, and secondary factors like resilience, gumminess, and chewiness, were the major factors measured.

#### 2.2.4. Water Activity of Raw Chicken Meat

Water activity (aw) values of raw chicken meat were measured using an AQUALAB 4TE (Fort Collins, CO, USA) device. Meat samples were placed in the container up to the marking and placed in the device for the reading of the aw values [19].

#### 2.2.5. Determination of Drip Loss in Raw Chicken Meat

The weight difference between the raw meat sample that was taken and the raw meat sample that was stored (for 24 h at 4 °C in plastic bags and for less than 5 h postmortem) was used to calculate drip loss [20]. The percentage of the weight difference was used to compute drip loss.

### 2.3. Meat Metabolite Extraction

Raw broiler chicken meat (fifty grams) was used individually for each bacterial inoculum. Metabolite extraction was carried out with modification in the methods described by González-Peña et al. [21]. The frozen chicken meat of all the inoculated and control samples with replications (stored at −80 °C in liquid nitrogen) were homogenized individually to a fine powder in liquid nitrogen using a pestle and mortar. The powder (500 mg) and cold methanol (1 mL) were mixed on a vortex mixer for 10 s. The resultant mixture was immersed in ice for 10 min, vortex mixing for 10 s, and later centrifuged at 14,000× *g* rpm for 10 min (at room temperature). The supernatant was collected in 1.5 mL centrifuge tubes (Eppendorf) and stored at −80 °C before analyzing by FTIR and GCMS.

#### 2.3.1. Meat Metabolite Profiling and Fingerprinting

##### GCMS Analysis

The pure methanolic extract was subjected to GCMS analysis as per Zahari et al. [22] (Gas chromatograph Clarus^®^, 680 and Mass spectrometer Clarus^®^ SQ8C, Shelton, CO, USA). The GCMS studies were carried out using a capillary column (30 m × 0.25 m). The oven was set at a temperature of 40 °C for 2 min, followed by a 10 °C min^−1^ run at 250 °C, held on for 2 min, and the detector temperature was set at 320 °C, with the input and source lines set at 230 °C. Helium (He) was used as a carrier gas at a linear flow rate of 20 cm s^−1^. The scanning mode of the MS technique was chosen, with a solvent delay of 0.00 to 0.50 min. The GC approach was selected with a 20 min run duration. The MS detector was set to 200 °C, and the meat metabolites found were identified using a mass spectral database National Institute of Standards and Technology Library (NIST).

##### FTIR Analysis

Spectra of the metabolite extracts of control and spiked sample were recorded in the wavelength range of 4000–400 cm^−1^ by FTIR (FT/IR-6800 type A endowed with ATR PRO ONE, Tokyo, Japan) following the method of Signorini et al. [23].

### 2.4. Statistics

All the collected data of physical parameters and FTIR spectral data were analyzed with R software, version 4.3.1, Auckland, NZ and OriginLab software, 2017, 9.4, (Northampton, NC, USA). The obtained GCMS data were copied to Microsoft Excel and then pathway and network analysis of the data was performed by using Cytoscape, version 3.10.2 (Seattle, WA, USA) and MetaboAnalyst 6.0 software (Alberta, CA, USA).

## 3. Result and Discussion

### 3.1. Physical Quality Parameters of Collected Raw Chicken Meat Sample

Prior to the start of the experiment, the meat quality parameters such as pH, color, texture, drip loss, and water activity were assessed to know about the physical quality of collected meat samples.

#### 3.1.1. Effect of pH

The pH factor had a direct relationship with the microbial load, shelf life, and metabolic activity of meat and is considered as one of the important quality parameters of raw meat [23,24]. The increase in the pH of raw chicken meat spiked with foodborne pathogens is likely due to the production of ammonia by the microorganisms. Specifically, the pH levels were observed to rise from 5.95 ± 0.18 before treatment (Table S1) to 6.35 ± 0.11 for *E*. *coli* O157:H7, 6.08 ± 0.10 for *P*. *aeruginosa*, 6.21 ± 0.09 for *Salmonella*, and 6.15 ± 0.11 for *Staphylococcus*. This shift in pH suggests that metabolic byproducts, such as ammonia, generated by the pathogens influence the meat’s pH levels. This is due to glucose exhaustion by such spoilage organisms. It is well documented that raw meat at normal pH and room temperature is more susceptible to spoilage and within two hours, the change in organoleptic characters can be detected [25]. The increased pH of raw chicken meat makes it more available for further survival, as many of the foodborne pathogens cannot thrive under lower pH conditions [26].

#### 3.1.2. Color Value of the Collected Sample

**Figure 1:**
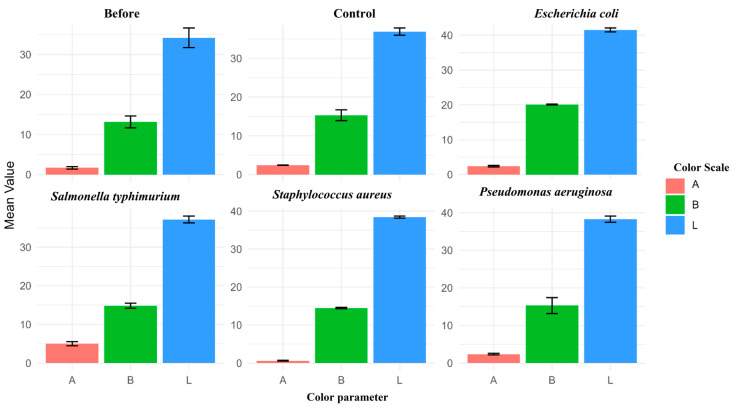
Effect of different treatments on color values of meat samples. Bar graphs show the mean values of color parameters: A, B, and L across treatments including Before, Control, *E*. *coli*, *Salmonella*, *Staphylococcus*, and *Pseudomonas*. Error bars represent the standard error of the mean (SEM) calculated from replications (biological and technical). Statistically significant differences between treatments were determined using one-way ANOVA (A: F (5,12) = 28.04, *p* < 0.001; B: F (5,12) = 3.699, *p* = 0.0294; L: F(5,12) = 3.998, *p* = 0.0229). Significant differences were observed in all three parameters (A: Red/green, B: Blue/yellow, and L: Lightness) across treatments, with higher significance in A values (*p* < 0.001).

The observed color values are consistent with the studies conducted by Wattanachant et al. [27] and Davoodi et al. [28]. The color values L*, A*m and B*, increased after two hours (Figure 1) of treatment, and the samples inoculated with pathogens showed more change than that of the control and before treatment values.

This change or increase in color values is due to the occurrence of degradation by the enzymes and metabolites produced by the spiked foodborne pathogens. Also, the metabolites produced due to lipid oxidation will oxidize myoglobin and this plays a major role in meat discoloration.

#### 3.1.3. Texture Profiling of the Collected Sample

Texture profiling of raw meat sample at the time of collection and after two hours of incubation of control and spiked sample projected significant differences over hardness, fracturability, springiness, resilience, cohesiveness, adhesiveness, and chewiness except for the attribute gumminess (Table S2). The highest hardness (44,303.8 g) and fracturability (40,663.3) were observed in the fresh meat sample, i.e., before treatment. Hardness gives information on the maximum amount of force required for deformation [29]. The minimum hardness observed in the spiked sample (12,693.01 g in the *Salmonella*-spiked sample) might be due to continued exposure of the sample to foodborne pathogens causing protein denaturation. Fracturability was nil in the control and spiked sample two hours later due to the loss of tensile strength due to protein denaturation. The springiness value indicates how long it takes for the material to restore its shape and texture following compression [30]. Hence, the highest springiness was reported in the sample spiked with *Pseudomonas* (0.79) indicating that the sample was more prone to deformation with high viscosity. Likewise, cohesiveness (0.094), adhesiveness (11.047), and chewiness (11,868.656) were found to be higher in the *Pseudomonas*-spiked sample, as cohesiveness gives information on elasticity, adhesiveness on stickiness, and chewiness on food mastication [31].

#### 3.1.4. Water Activity of Raw Chicken Meat

Water activity is a crucial factor for microbial growth and survival. It also helps in predicting the shelf life and product quality [32]. Fresh chicken meat samples recorded higher water activity (0.986) as compared to all other pathogen-spiked samples. Foodborne pathogens interacting with carcasses will metabolize nutrients in them. These pathogens will consume water as they grow and multiply, leading to decreased water activity. Acid secretion by these pathogens will also lower the water activity.

#### 3.1.5. Drip Loss in Raw Chicken Meat

Drip loss gives information on water holding capacity; where drip loss increases, water holding capacity decreases [33]. Fresh meat samples reported a very lower drip loss of 0.18%, whereas spiked samples reported increased drip loss percent. This higher drip loss of 4.58 in the *Pseudomonas*-spiked sample (Supplementary Table S1) indicates poor quality, as it results in lower sensory parameters such as such as toughness, dryness, and juiciness of the sample.

### 3.2. Meat Metabolite Profiling and Fingerprinting

#### 3.2.1. GC-MS Analysis

**Figure 2:**
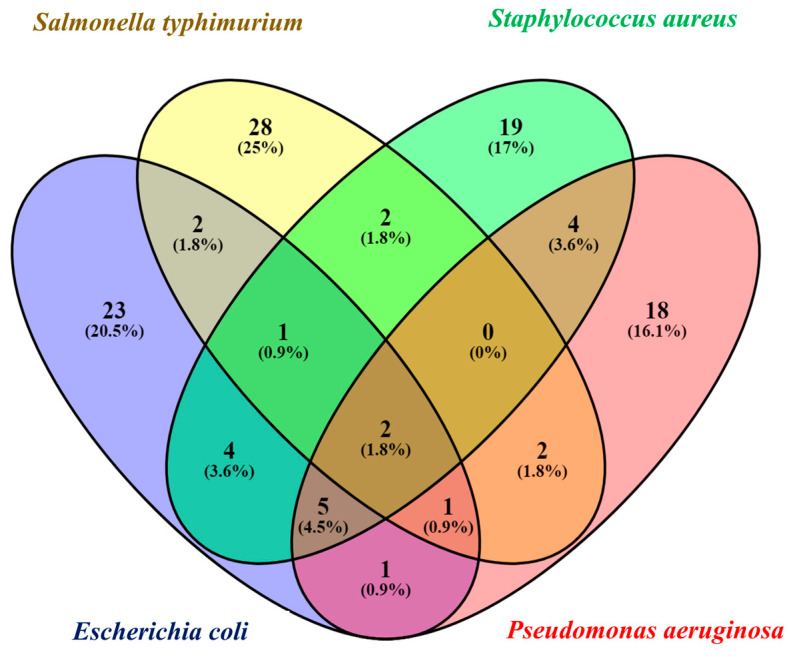
Venn diagram of total identified metabolites of control and spiked raw chicken meat sample.

Untargeted metabolite profiling was carried out by extracting metabolites from control and spiked raw chicken meat samples. The main objective of the study was to identify biomarkers for detecting foodborne pathogens by discerning the metabolites produced by control and spiked samples. Metabolomics has the potential to identify a wide range of metabolites present in meat and the associated pathogens. This helps in determining the potential biomarkers, which can be further applied for the rapid detection of foodborne and spoilage pathogens [34]. A total of 113 metabolites were identified from all the samples. In total, 23 metabolites for *E*. *coli* O157:H7, 28 metabolites for *Salmonella*, 19 metabolites for *Staphylococcus,* and 18 metabolites for *Pseudomonas* were identified. Figure 2 shows an illustration of the total identified metabolites using Venn diagram.

**Figure 3:**
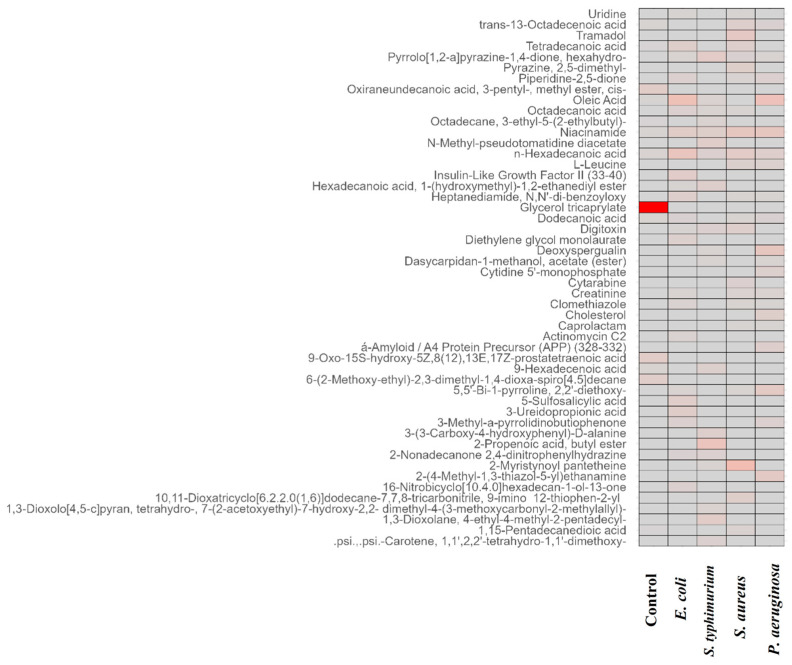
Heat map analysis of selectively identified metabolites using R software.

Pathway analysis of the obtained GCMS data revealed that most of the identified metabolites contributed more to the fatty acid pathway such as saturated fatty acid beta-oxidation pathway (Figure S1) and the metabolites of the spiked sample contributed more to amino acid degradation pathways such as valine, leucine, and isoleucine degradation (Figure S2). Apart from this, metabolites identified were involved in pathways such as glycosphingolipid metabolism, linoleate metabolism, lipoate metabolism, lysin metabolism, methionine and cysteine metabolism, pyrimidine metabolism, cholesterol biosynthesis, urea cycle, arginine, proline, glutamate, aspartate and asparagine metabolism, and vitamin B3, B9, and H metabolism was identified using Metscape analysis of Cytoscape. Further analysis of the identified metabolites was carried out using MetaboAnalyst 6.0 software, where the dot plot (Figure S3) of enrichment analysis of the metabolites identified from spiked samples confirmed that the identified metabolites were affirmed more with fatty acid metabolism pathways. Fatty acids are the primary compounds in the membrane structure of bacteria; hence this affirms that the microorganisms break down fatty acids and utilize them as a major energy source for their nutrition and growth. Glycerol tricaprylate, a medium chain triglyceride found in lipids, found to be predominant in control, was observed in heat map analysis (Figure 3), whereas it was observed very little or absent in the spiked meat sample, which is due to the beta oxidation of saturated fatty acids that results in degradation of such metabolites [35].

Fatty acids such as Hexadeconoic acid, oleic acid, dodecanoic acid, tetradeconoic acid, trans-13-Octadecenoic acid, and 1,15-Pentadecanedioic acid were involved in the saturated fatty acid beta oxidation pathway and these fatty acids were already found to be reported by few researchers [36,37]. Unique metabolites identified upon the interaction of raw chicken meat and foodborne pathogens could be identified as potential biomarkers in detecting the presence of foodborne pathogens. The metabolites such as ureidopropionic acid, 5-sulfosalicylic acid, 11,14-eicosadienoic acid, methyl ester for *E*. *coli* O157:H7; 11-bromoundecanoic acid, neocurdione, glafenin, and eicosanoic acid for *Salmonella*; azepan-1-yl-acetic acid, methyl ester, tramadol, cytarabine, and dipipanone for *Staphylococcus*; and cyclopentane undecanoic acid, phosphonofluoridic acid, and î-n-formyl-l-lysine for Pseudomonas could be identified as potential biomarkers upon meat metabolite profiling for the presence of foodborne pathogens. The quality of meat relies on uracil which contributes to the formation of β-alanine, a precursor for carnosine [38]. The presence of the ureidopropionic acid compound identified in *E*. *coli* O157:H7-spiked sample exhibits the breakdown of uracil, a pyrimidine base and this states that ureidopropionic acid could serve as potential biomarker in identifying the quality of meat. The other major metabolite, 11,14-eicosadienoic acid is a polyunsaturated fatty acid (PUFA) with lower oxidative stability. Upon oxidation, PUFA can lead to the development of undesirable flavor compounds in meat, this can also act as a significant compound in influencing both the nutritional and sensory qualities of the meat [3]. In the present study, 11,14-eicosadienoic acid has been found in both *E*. *coli* O157:H7 and *Salmonella*-spiked samples, which could have influenced meat flavor. However, the compound glafenin found in *Salmonella*-spiked samples could be a potential detectable marker and it was reported to cause severe gut abnormalities in zebrafish [37]. The oxidative modification of protein in meat was confirmed with the presence of î-n-formyl-l-lysine, which might be due to protein aging or oxidative stress [39]. The presence of î-n-formyl-l-lysine in the *Pseudomonas*-spiked meat sample confirmed the oxidative damage in the meat sample which indirectly might affect the quality of meat. Hence, î-n-formyl-l-lysine could serve as a potential biomarker for the rapid detection of pathogens.

**Figure 4:**
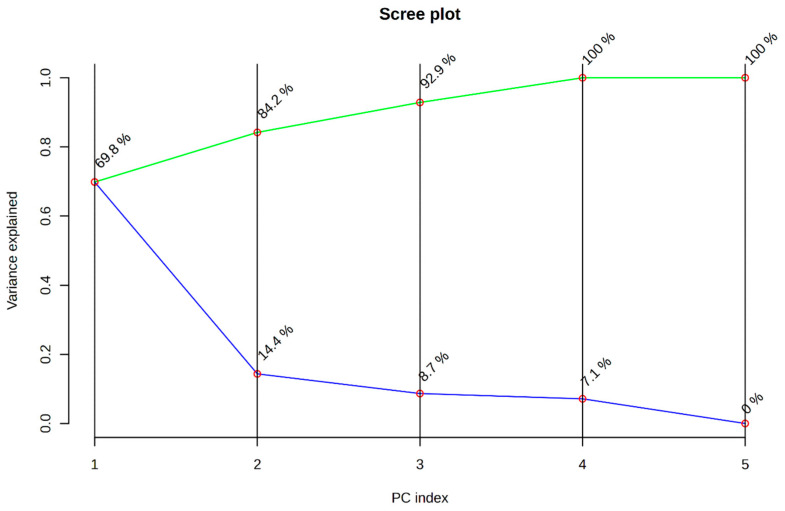
PCA analysis of similar metabolites of all five samples—Scree plot analysis with cumulative PC variance (green line) and individual PC variance (blue line).

All other corresponding unique metabolites were not identified either in the control or between the spiked samples. Principle component analysis (PCA) was performed for the metabolites niacinamide, dodecanoic acid, dodecanoic acid, n-Hexadecanoic acid, oleic acid, and pyrrolo (1,2-a) pyrazine that were similar in both control and spiked samples analysis. The scree plot of PCA analysis (Figure 4) reported the existence of cumulative variance and individual variance (PC1-69.8%) and also the scattered 3D plot (Figure S4) displayed higher variation in the control than in the spiked sample, making the detection more specific and sensitive. Thus, meat metabolomics holds great potential for quality analysis and authentication, enabling comprehensive characterization of complex meat matrices. Despite successful research identifying metabolomic profiles and biomarkers, its adoption for routine analysis and meat surveillance remains limited [40]. Further investigation on these metabolites is required to understand the mechanisms behind each metabolite that could act as a potential biomarker.

#### 3.2.2. FTIR Analysis—Spectral Fingerprinting

**Figure 5:**
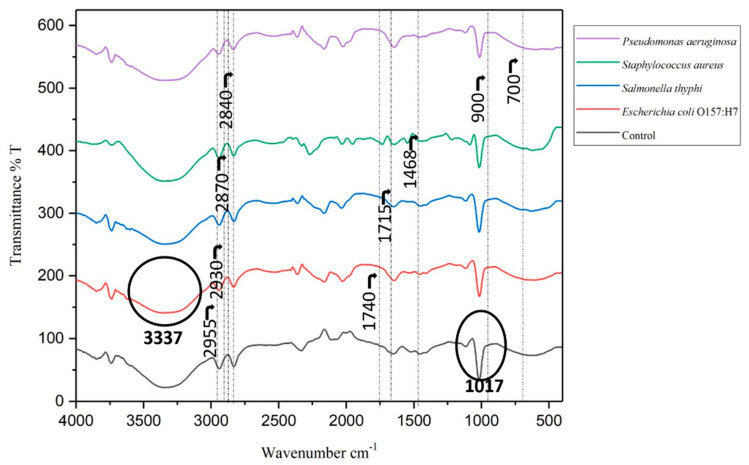
FT-IR spectra (400–4000 cm^−1^) of five samples Control, *E*. *coli* O157H7, *Salmonella typhimurium*, *Staphylococcus aureus*, and *Pseudomonas aeruginosa*.

FTIR is already used widely in the identification and characterization of bacteria, now its application is extended in ensuring food safety by early detection and classification of foodborne pathogens that are intact with the food matrix based on the spectral fingerprinting that is reproducible by following a simple sample preparation procedure [41,42]. FTIR analysis was carried out on an FT/IR-6800 type A endowed with ATR PRO ONE using a triglycine sulphate detector under a standard light source. Methanolic extracts of control and spiked samples after two hours of aerobic incubation at 37 ± 2 °C were subjected to FTIR analysis. The spectral analysis was carried out between 4000 and 400 cm^−1^ with a spectral resolution of 4 cm^−1^. Though pathogens share the same biochemical components such as proteins, polysaccharides, phospholipids, and nucleic acids, their quantities and distribution vary between strains, resulting in a unique FT-IR spectrum for each pathogen [43,44,45]. The graphical illustration of spectral fingerprinting corresponding to the control and the spiked sample is presented in Figure 5 and the functional group corresponding to the observed peaks is presented in Table 1.

A higher peak intensity was observed at 1017 cm^−1^ corresponding to the glycosidic bond [25,46] connecting carbohydrates with other groups of compounds, and a decreased peak intensity at 1017 cm^−1^ was observed in the spiked sample in comparison with the control. This shows evidence of the breakdown of carbohydrates into sugars by foodborne pathogens. The clear-cut peak at 3337 cm^−1^ corresponds to O-H bond of primary aliphatic amines [47], which is comparably reduced in the case of the spiked sample than in control because the metabolites produced by the pathogens may create unstable and changeable conditions [48].

**Table 1:** Significant FTIR spectral peaks and functional group variations across control and pathogen-inoculated meat samples [12,49,50].

Wavenumber (cm^−1^)	Functional Group	Corresponding Bioorganic Compounds	Control	*E. coli* O157	*S. typhimurium*	*S. aureus*	*P. aeruginosa*
3337	O-H bond	Aliphatic amines	Present	Decreased	Decreased	Slightly increased	Decreased
2957–2953	Asymmetric CH_3_ stretching	Lipids	Present	Decreased	No change	Increased	Decreased
2930	C-H stretching	Alkane	Present	No change	Slightly increased	No change	Slightly increased
2875–2870	Symmetric CH_3_ stretching	Lipids	Present	Decreased	No change	Slightly increased	Decreased
2840	C-H stretching	Alkane	Present	Decreased	Slightly increased	No change	Decreased
1740	C=O stretching	Ketones	Present	Increased	Increased	Decreased	Increased
1715	C=O stretching	Nucleic acid and carbonic acid	Present	Increased	No change	Decreased	Increased

The peak corresponding to the wavenumber 2957–2953 cm^−1^ was assigned to CH_3_ asymmetric stretching, the peaks at 2875–2870 cm^−1^ were assigned to symmetric CH_2_ stretching related to the lipid molecules where the spectral peak was distinct from the control in the case of *Pseudomonas,* and the peaks at 1715 cm^−1^ belong to C=O carbonyl stretching relating to cholesterol and triglyceride esters showing noticeable peak shift in *E*. *coli* O157:H7-spiked sample (Table 1). The wavenumber 1600 to 700 cm^−1^ was observed to have a mixed composition of fatty acids and lipids contributing more to the bacterial identification [51]. The GCMS report has already shown the presence of more metabolites belonging to fatty acids. The change observed in the fatty acid spectral region could be taken as a fingerprinting spectrum for the detection of pathogens upon further research. Principle compound analysis (PCA) of the obtained spectral data showed significant variance among the control and spiked samples. Data analysis resulted in a significant eigenvalue of 4.42 for PC1 with 88.59% of total variance. The loading plot of PCA analysis (Figure S5) showed that PC1 had heavy loadings with variation among the spectral data of *Salmonella*, *E*. *coli* O157:H7 and *Pseudomonas*, whereas PC2 showed heavy loading for the spectral data of the control and *Staphylococcus*. These findings suggest that FTIR, combined with PCA analysis, holds promise for differentiating pathogenic microorganisms based on their unique biochemical changes, particularly in carbohydrate, lipid, and fatty acid regions. Additionally, there are some advanced systems like optical biosensors, which were used in a study conducted by Mura et al. [52], which detected pathogenic *E*. *coli* O157:H7 using FTIR spectroscopy and resulted in mid-infrared fingerprints of pathogens in buffer. These fingerprints arise from functional groups associated with proteins, lipids, and carbohydrates. MIR spectra enable pathogen identification and structural characterization, distinguishing between pathogens and subspecies. MIR spectra are sensitive and additive, allowing for the fingerprinting and quantification of the target pathogen, thus enhancing traditional devices into highly sensitive biosensing systems. Hence, FTIR fingerprints of control and spiked samples showed that spectral analysis could be used for partial detection of pathogens with a strong accentuation that still more deliberations are needed for creating more specific and sensitive methods of detection of foodborne pathogens. Further research could refine this approach as a reliable pathogen detection tool.

## 4. Conclusions

In the current study, our results showed that raw chicken meat samples spiked with foodborne pathogens were subjected to changes in their physical parameters such as pH, color, texture, drip loss, and water activity. An increase in the pH of the spiked sample was observed, which might be due to the production of ammonia by microorganisms. The color values of spiked samples showed significant differences over the control. The discoloration of the sample might be due to the degradation of compounds by the enzymes produced by inoculated pathogens. Texture profiling of the treated meat sample was analyzed and a lower hardness was reported in *Salmonella*-spiked meat than in the control, due to denaturation of protein caused by the spiked pathogens. Water activity and drip loss of the treated raw meat sample were also analyzed and were found to have a deviation from the control sample.

Control and spiked raw chicken meat samples were subjected to GCMS and FTIR analysis to identify potential biomarkers and spectral fingerprinting to assess the quality of meat. Moreover, the metabolites identified through GCMS analysis such as ureidopropionic acid and 11,14-eicosadienoic acid, in *E*. *coli* O157:H7; 11-bromoundecanoic acid, glafenin, and eicosanoic acid for *Salmonella*; and cyclopentane undecanoic acid, and î-n-formyl-l-lysine for *Pseudomonas* revealed the bioactive molecules/biomarkers that belong to fatty acids and amino acid degradation pathways contributed to the identification of potential biomarkers. FTIR spectral data revealed substantial variance in the spectral pattern of control and spiked samples and showed significant discrimination upon fine data analysis that could be used for partial detection of foodborne pathogens. Hence, variations were observed among all the samples in both GCMS and FTIR analysis, enabling more specific detection of individual pathogens.

Based on the biomarkers and spectral pattern identified in the current study, future research could be carried out to focus on the development of a rapid and field-level kit for the detection of foodborne pathogens that could utilize simple chemical or spectral tests to provide quick and accurate result by GCMS and FTIR to ensure microbial food safety in production and processing plants of meat. Further, these findings could be integrated with artificial intelligence (AI) and machine learning (ML) to develop systems that continuously monitor meat quality and detect pathogens in real-time. The findings could also help in strengthening food safety regulations by identifying biomarkers linked to pathogens.

## Data Availability

The original contributions presented in the study are included in the article. Further inquiries can be directed to the corresponding author.

## Supplementary Materials

The following supporting information can be downloaded at: https://www.mdpi.com/article/10.3390/foods13213416/s1, Figure S1: Saturated fatty acid beta-oxidation pathway by Metscape pathway analysis using Cytoscape software; Figure S2: Valine, leucine, and isoleucine degradation pathway by Metscape pathway analysis using Cytoscape software; Figure S3: Dot plot of enrichment analysis for the metabolites of the control and spiked samples; Figure S4: Synchronized 3D plot of peak area percent of similar metabolites identified in all the five samples Red—Control, Green—E. coli O157H7, Blue—Salmonella, Pink—Staphylococcus, Dark blue—Pseudomonas; Figure S5: Loading plot of PCA analysis for FTIR spectral data of the control and spiked samples; Table S1: Physical parameters such as pH, water activity, color value, and drip loss of the control and spiked samples; Table S2: Texture profiling of the control and spiked samples; Table S3. Specific primers used to check the purity of the reference strains.
