# Supplementary material for: Identification of Potential Biomarkers and Spectral Fingerprinting for Detection of Foodborne Pathogens in Raw Chicken Meat Matrix Using GCMS and FTIR
Source: Foods. 2024 Oct 26;13(21):3416. doi: 10.3390/foods13213416 (PMC11545171; doi:10.3390/foods13213416)

Table S1. Physical parameter such as pH, Water activity color value and drip loss of control and spiked sample

Treatment	pH	Water activity	Color value			Drip loss %
			L	A	B	
Before	5.95 ± 0.18^{c}	0.986±0.009^{a}	34.16±4.23^{c}	1.70±0.52^{b}	13.16±2.57^{b}	0.18
Control – A	5.73 ± 0.01^{d}	0.975±0.003^{b}	36.90±1.60^{bc}	2.43±0.05^{b}	15.30±2.40^{a}	1.29
E coli	6.35 ± 0.11^{a}	0.962±0.007^{c}	41.53±0.94^{a}	2.40±0.34^{b}	20.10±0.20^{b}	2.51
Pseudomonas	6.08 ± 0.10^{bc}	0.963±0.002^{c}	38.30±1.45^{ab}	2.36±0.35^{b}	15.26±3.69^{b}	4.58
Salmonella	6.21 ± 0.09^{ab}	0.956±0.006^{c}	37.20±1.53^{bc}	5.00±0.88^{a}	14.83±1.13^{b}	4.42
Staphylococcus	6.15 ± 6.15^{abc}	0.974±0.004^{b}	38.46±0.47^{ab}	0.60±0.17^{c}	14.46±0.28^{b}	2.48

*Mean ± SD calculated with three replications

Table S2. Texture profiling of control and spiked sample

Treatment	Hardness (g)	Fracturability	Springiness	Resilience	Cohesiveness	Adhesiveness	Chewiness
Before	44303.80±1794.58^{a}	40663.30±1086.81^{a}	0.32±0.01^{c}	0.141±0.003^{a}	0.149±0.004^{d}	-27.271±0.550^{c}	2401.846±133.046^{b}
Control – A	38028.96±1308.15^{c}	0.0±0.0^{b}	0.34±0.02^{c}	0.122±0.004^{b}	0.240±0.017^{c}	-26.157±0.799^{c}	1719.786±63.559^{c}
E. coli	40499.93±796.37^{b}	0.0±0.0 ^{b}	0.18±0.01^{d}	0.093±0.007^{c}	0.123±0.006^{e}	-23.513±0.852^{b}	880.593±4.938^{d}
Pseudomonas	33191.89±897.30^{d}	0.0±0.0 ^{b}	0.79±0.03^{a}	0.094±0.006^{c}	0.427±0.022^{a}	-11.047±2.232^{a}	11868.656±602.507^{a}
Salmonella	12693.01±806.29^{e}	0.0±0.0 ^{b}	0.39±0.01^{b}	0.129±0.004^{b}	0.265±0.014^{b}	-33.96±1.084^{d}	1302.260±42.356c^{d}
Staphylococcus	13303.81±559.95^{e}	0.0±0.0 ^{b}	0.33±0.01^{c}	0.129±0.002^{b}	0.253±0.007^{bc}	-35.951±0.836^{d}	1132.233±25.936^{d}

*Mean ± SD calculated with three replications

Figure S1 Saturated fatty acid beta-oxidation pathway by Metscape pathway analysis using Cytoscape software

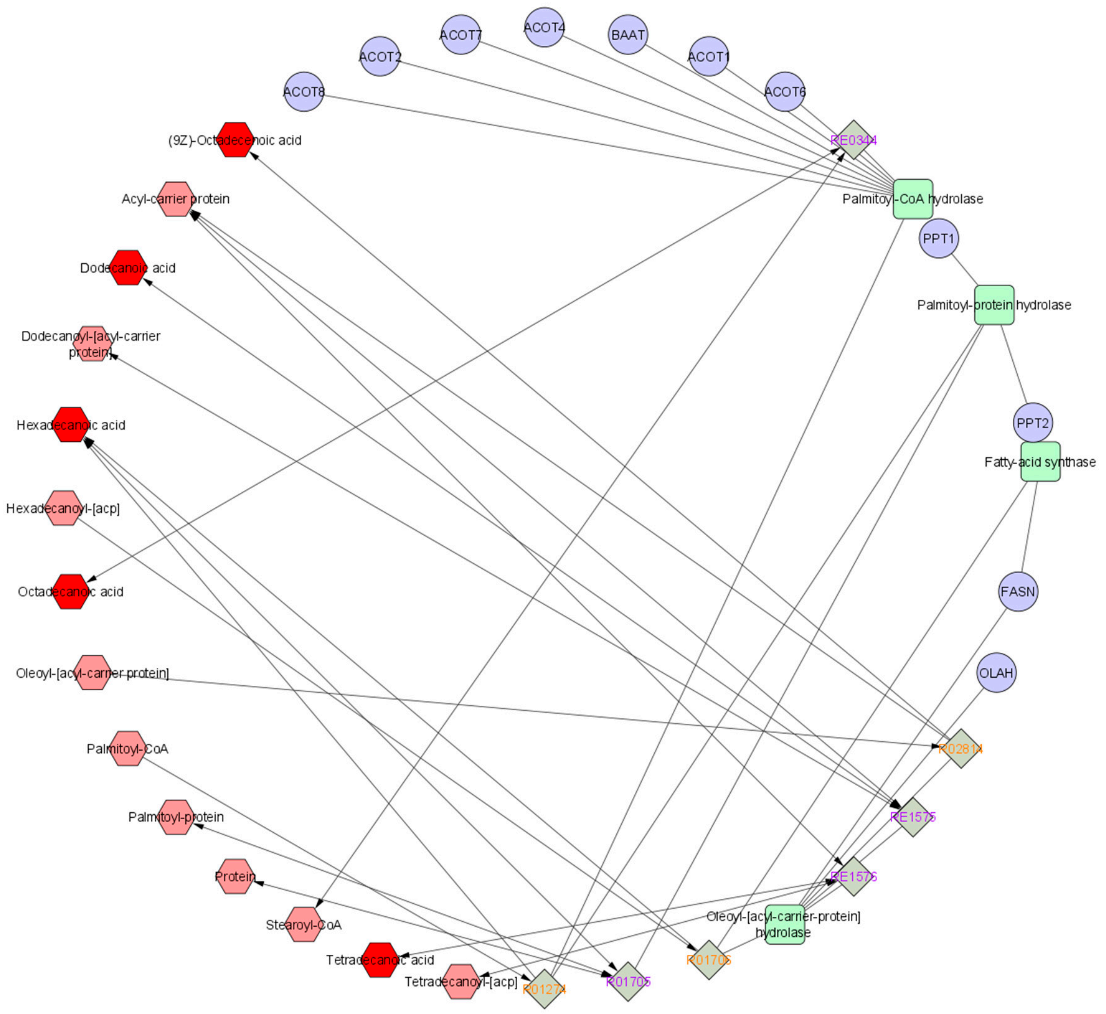

Figure S2 Valine, leucine and isoleucine degradation pathway by Metscape pathway analysis using Cytoscape software

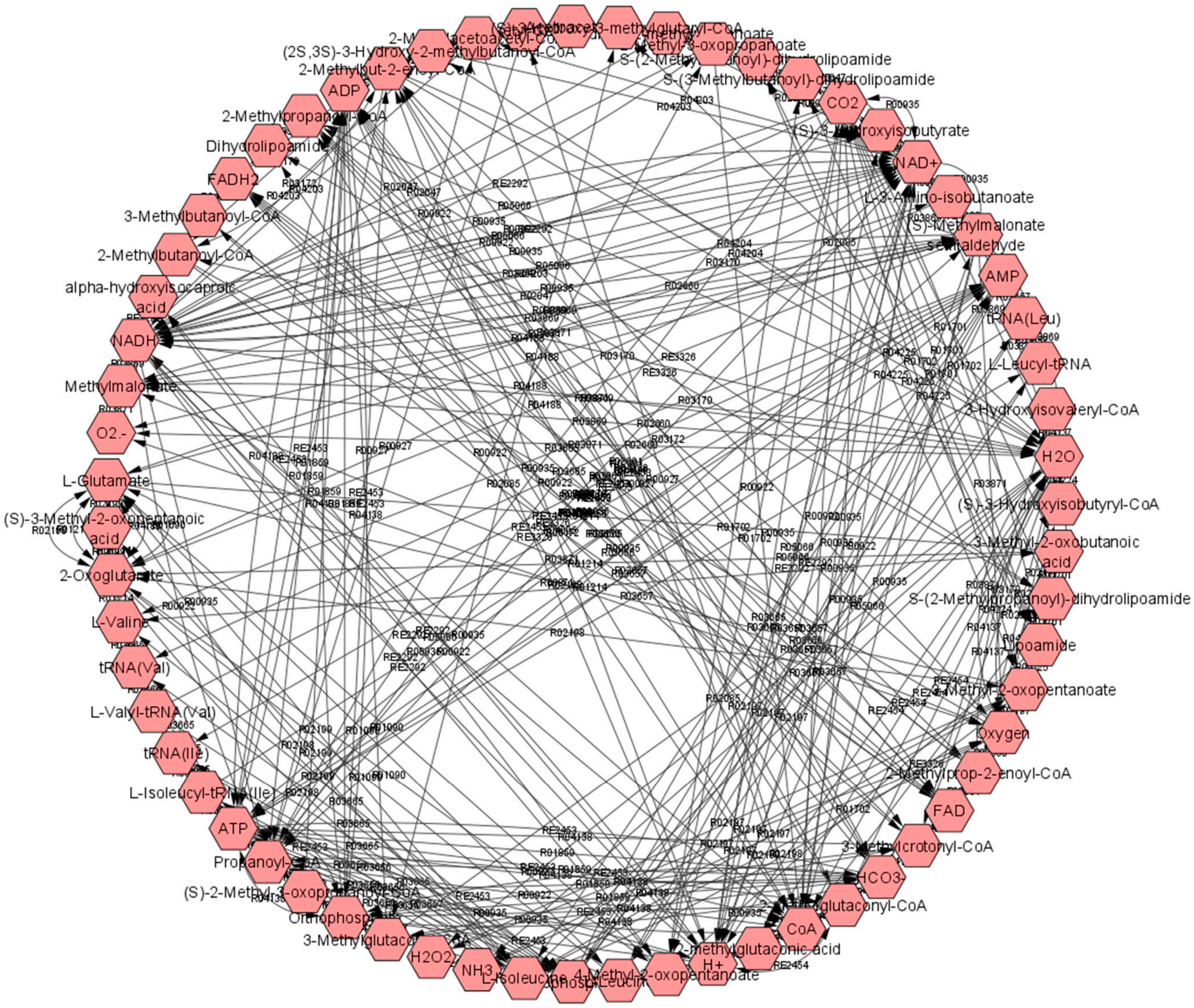

Figure S3 Dot plot of enrichment analysis for the metabolites of control and spiked sample

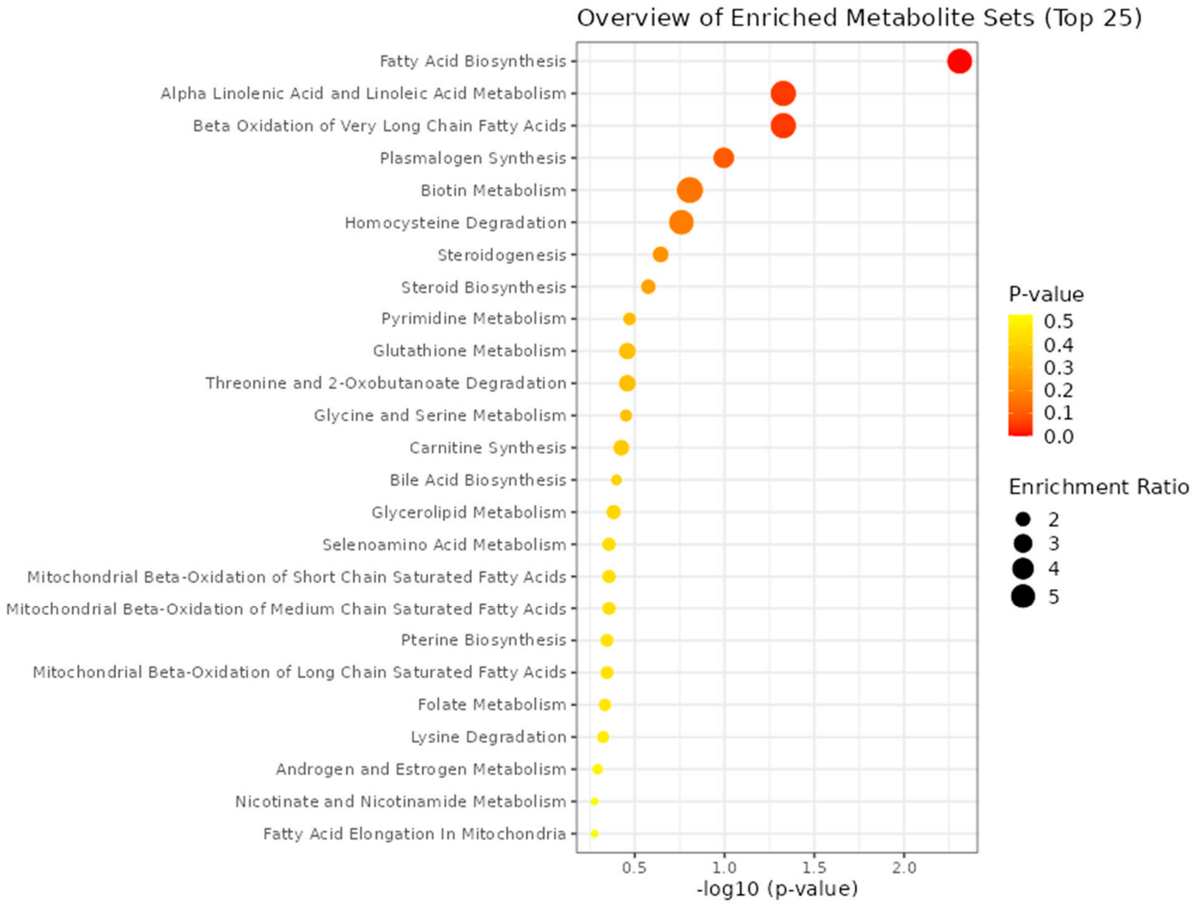

Figure S4 Synchronized 3D plot of Peak area percent of similar metabolites identified in all the five samples Red -Control, Green - *E. coli* O157H7, Blue - *Salmonella*, Pink - *Staphylococcus*, Dark blue - *Pseudomonas*

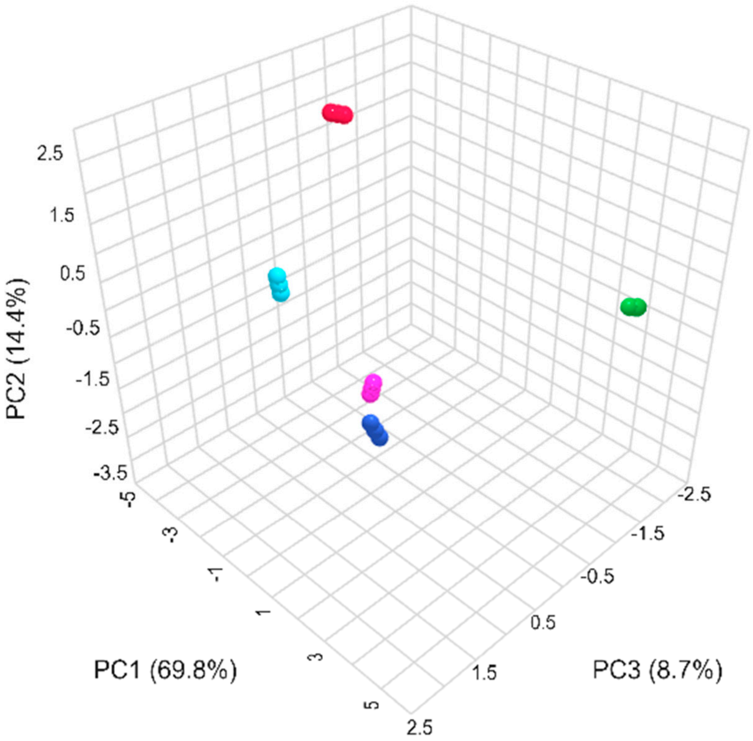

Figure S5 Loading plot of PCA analysis for FTIR spectral data of control and spiked sample

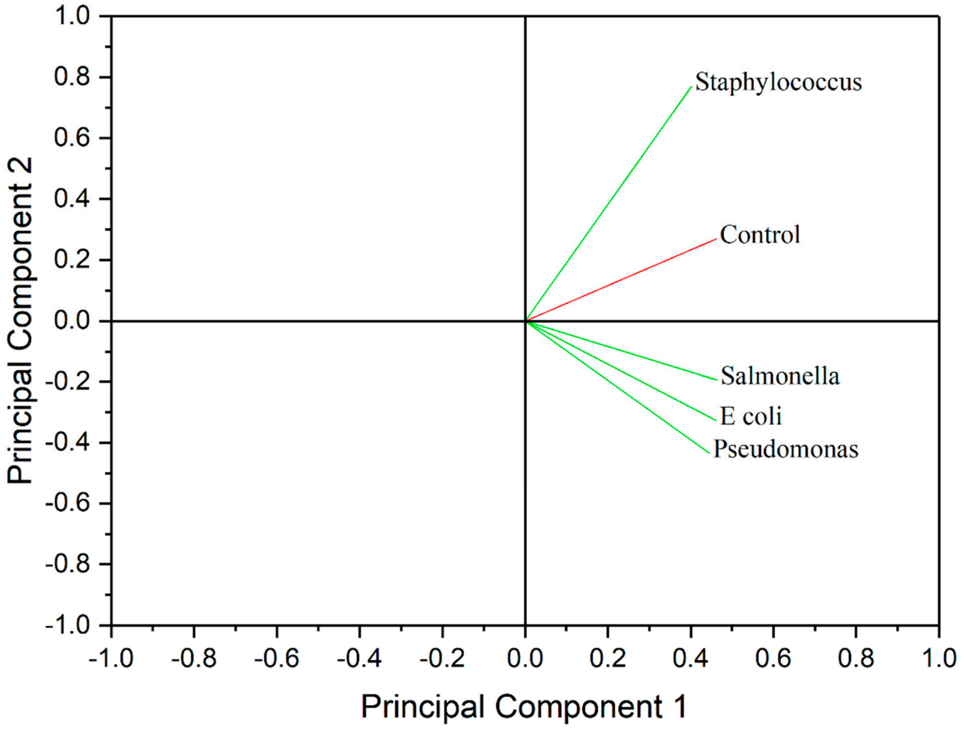

Supplement: Supplementary file 1 [file foods-13-03416-s001.zip › Supplementary file.pdf]
